# Effects of non-surgical periodontal therapy on periodontal clinical data in periodontitis patients with rheumatoid arthritis: a meta-analysis

**DOI:** 10.1186/s12903-021-01695-w

**Published:** 2021-07-10

**Authors:** Yu Huang, Zheng Zhang, Youli Zheng, Zhulan Zhao, Yang Zhong, Qingyu Zhang, Degeng Xia, Ning Ma, Li Zhang

**Affiliations:** 1 Hospital of Stomatology, Jilin University, 1500th Qinghua Road, Changchun, 130021 Jilin China; 2 School of Medicine, Tianjin Stomatological Hospital, Nankai University, Tianjin, 300041 China; 3 Tianjin Key Laboratory of Oral and Maxillofacial Function Reconstruction, Tianjin, 300041 China; 4 Hospital of Stomatology, Tianjin Medical University, Tianjin, 300070 China

**Keywords:** Periodontitis, Rheumatoid arthritis, Scaling and root planing, Meta-analysis

## Abstract

**Backgrounds:**

To date, there is still no consensus about the clinical efficacy of non-surgical periodontal therapy in rheumatoid arthritis (RA) patients with periodontitis. Therefore, the aim of this study was to summarize clinical data regarding the efficacy of scaling and root planing (SRP) in patients with RA and periodontitis compared to non-RA periodontitis patients.

**Methods:**

We selected randomized controlled trials (RCTs) that compared periodontal clinical data in RA as compared to non-RA periodontitis patients by searching Embase, PubMed and Cochrane Central Register of Controlled Trials and by manually retrieving from the earliest records to March 8, 2021. The overall effect size of plaque index (PI), gingival index (GI), attachment loss (AL), probing depth (PD) and bleeding on probing (BOP) were calculated by either a fixed or random-effect model, and subgroup analyses were conducted according to the different time points of follow-up. Two investigators extracted the data and assess the accuracy of the obtained results with 95% of Confidence Intervals (CI). Cochrane Collaboration's tool was responsible for the evaluation of the literature quality and the inter-study heterogeneity was evaluated by Q test and I^2^ statistic. Sensitivity analyses were applied for results with heterogeneity. Publication bias was determined by Begg's test, Egger's test and the trim-and-fill method.

**Results:**

Seven RCTs including 212 patients eventually met the inclusion criteria for the study. As the primary results, the change of PD was not statistically significant and in the secondary results changes of PI, GI, AL and BOP were also not statistically significant in RA patients with periodontitis compared to non-RA periodontitis patients. In subgroup analysis, a larger BOP reduction at 3 months, PI and AL reduction at 6 months were observed in patients with RA and periodontitis group. The results of sensitivity analyses had no significant effect. No evidence of potential publication bias was tested. There were some limitations due to the small number of eligible RCTs.

**Conclusions:**

SRP is equally effective in RA as compared to non-RA periodontitis patients. It suggests RA does not affect the clinical efficacy of non-surgical periodontal therapy. These results could serve evidence-based practice.

**Supplementary Information:**

The online version contains supplementary material available at 10.1186/s12903-021-01695-w.

## Background

Periodontitis is a chronic inflammation of the periodontal tissues, with negative impact on both local and systemic health. It is well known that the inflammatory state gives rise to a multitude of damage of periodontal tissue, of which the most critical are in alveolar bone, as well as in periodontal ligament [1, 2]. In a comprehensive epidemiological report in 1990 and 2010 of severe periodontitis (SP), a global age-standardized rate of severe periodontitis was reported to be high around 11.2% [3]. It suggested a growing global health threat from severe periodontitis. In addition, many modifiable and non-modifiable risk factors, such as rheumatoid arthritis (RA), diabetes, obesity, high blood pressure, atherosclerosis and other cardiovascular diseases and so on, can modify the individual's risk of developing periodontitis, as well as the response to periodontal therapy [4–8].

RA is a chronic autoimmune disorder and can ultimately lead to the irreversible damage to cartilage in joints and loss of function even, which is closely related to the production of autoantibodies, synovial inflammation and hyperplasia [9, 10]. The interplay between RA and periodontitis has long been studied, with evidence showing complex associations between these two distinct diseases [11, 12]. The pathogenesis of the two diseases are characterized by local destruction of hard and soft tissues as a consequence of inflammation [13, 14]. Additionally, there is strong evidence that people with RA have elevated risk for inflammation of periodontal ligament, respiratory mucosa and intestinal mucosa to some extent [15]. Studies among people with RA demonstrate significantly higher prevalence levels in patients with periodontitis [16–18]. To date, the mechanisms accounting for the aggravation of periodontitis by RA are not completely clarified.

The representative of non-surgical periodontal therapy as scaling and root planing (SRP) has been considered as the traditional treatment regime in managing periodontitis. Conventional clinical indices and parameters of periodontal health, namely plaque index (PI), gingival index (GI), attachment loss (AL), probing depth (PD) combined with bleeding on probing (BOP), are usually calculated to determine the efficacy of SRP.

In recent years, there have been several works discussing effects of periodontal treatment on RA markers [19–21]. In previous meta-analyses, Schilin Wen et al., Qingqin Tang et al. and Nicholas R Fuggle et al. evaluated the prevalence and periodontal parameters of periodontitis in RA patients [22–24]. Assessed by disease activity score, tender joint counts, swollen joint counts, visual analogical scale and C-reactive protein, a meta-analysis indicates that SRP could improve RA activity [25]. There were also meta-analyses examining the risk of periodontitis for RA [26, 27]. In a recent meta-analysis, the bidirectional relationship between periodontitis and RA was also analyzed [28]. Additionally, effect of SRP about the clinical activity and inflammatory markers in patients with periodontitis and RA was assessed in a systematic review[29]. However, to our knowledge, a comprehensive meta-analysis attempted to establish the clinical efficacy of SRP in terms of periodontitis parameters in periodontitis patients with RA has not yet emerged. In light of these considerations, meta-analysis is now imperative to assess the difference in the clinical efficacy of SRP between RA with periodontitis patients and non-RA periodontitis patients.

## Methods

### Focused question

In this meta-analysis, we followed the guidelines in accordance with the 2009 Preferred Reporting Items for Systematic Reviews and Meta-Analysis–PRISMA statement [30]. The PICO question was formulated as follows: “What is the efficacy, of non-surgical periodontal therapy with respect to periodontal clinical data in RA as compared to non-RA periodontitis patients?”

P (Population): RA patients with periodontitis;

I (Intervention): Non-surgical periodontal therapy;

C (Comparison): Non-surgical periodontal therapy in non-RA patients with periodontitis;

O (Outcome): Primary outcome, changes in clinical parameters, including PD; Secondary outcome, changes in clinical indices/parameters, including PI, GI, AL and BOP.

### Search strategy

Based on the PICO criteria, a search strategy was developed and executed using an electronic search. Online PubMed, Cochrane library and Embase from the earliest records to March 8, 2021 were systematically screened for the desired publications. Two investigators (Z Zhang and Y Huang) screened the titles, abstracts, and full articles independently according to eligibility criteria for study selection. The search strategy for PubMed and Cochrane library was: (((("Periodontitis"[Mesh] OR "Chronic Periodontitis"[Mesh] OR "Aggressive Periodontitis"[Mesh]) OR ("Periodontal Attachment Loss"[Mesh] OR "Periodontal Diseases"[Mesh] OR "Periodontal Pocket"[Mesh] OR "Alveolar Bone Loss"[Mesh])) OR "Tooth Loss"[Mesh]) AND ("Arthritis, Rheumatoid"[Mesh] OR "Arthritis, Juvenile"[Mesh])) AND (((((("Therapeutics"[Mesh] OR "therapy" [Subheading]) OR "Periodontal Debridement"[Mesh]) OR "Dental Scaling"[Mesh]) OR "Root Planing"[Mesh]) OR (("Periodontal treatment") OR ("Periodontal therapy"))) OR (((("Periodontal Index"[Mesh]) OR "Patient Outcome Assessment"[Mesh]) OR "Efficiency"[Mesh]) OR "Dental Plaque Index"[Mesh])). The search strategy for Embase was: (periodontitis OR chronic periodontitis OR aggressive periodontitis OR periodontal attachment loss OR periodontal diseases OR periodontal pocket OR alveolar bone loss OR tooth loss) AND (rheumatoid arthritis OR RA) AND ((therapeutics OR periodontal debridement OR dental scaling OR root planning OR periodontal treatment OR periodontal therapy) OR (periodontal index OR patient outcome assessment OR efficiency OR dental plaque index)). Additionally, hand search for references cited in the published original and review articles was also performed. We didn’t place any restrictions on the language of publications when searching these online databases and the unpublished works were not accounted.

### Eligibility criteria

The following study designs were included: (1) Type of study design must be randomized controlled trial (RCT); (2) The study subjects were RA with periodontitis; (3) Use of SRP as an intervention treatment; (4) Studies had a control group consisting of non-RA periodontitis patients who received SRP; (5) Changes at least in PD was recorded in the study; (6) follow-up of at least 1 months.

The excluded criteria for our study were: (1) The study design was not RCT; (2) Potential participants who had any other disease or combined with systemic antibiotic therapy; (3) Studies lacked of control group; (4) Studies did not record periodontal parameter of PD; (5) Articles where the full text and date was not available.

### Data extraction

The data extracted from each article by two investigators (Z Zhang and Y Huang) including following data: periodontal indices/parameters included in the results, first author, year of publication, location, sample size, gender and age, duration of RA, and time point of follow-up. A third researcher addressed all remaining discrepancies after consultation between the two investigators.

### Quality assessment

The quality of the RCTs was assessed in accordance with the Cochrane Collaboration's tool, including the following aspects of evaluations: (1) random sequence generation; (2) allocation concealment; (3) blinding of participants and personnel; (4) blinding of outcome assessment; (5) incomplete of the outcome data; 6.selective reporting and the other bias (i.e. non-objective therapy and completeness of follow up) [31]. Following the Cochrane Collaboration guidelines, each RCT was classified as being at low, unclear or high risk of bias.

### Statistical analysis

The differences (experimental minus control) of the changes (final values minus baseline values) were employed to calculate the net changes of PI, GI, PA, AL and BOP between the two groups. The pooled effect was expressed as mean difference (MD) with their associated 95% confidence intervals (CIs). It was defined as statistically significant if p-value was less than 0.05. Subgroup analyses were conducted according to the different time points of follow-up. The Cochran’s Q test and I^2^ statistic were employed to calculate heterogeneity among the included studies. *P* < 0.05 (Q test) or I^2^ > 50% represented a substantial high level of heterogeneity, the random-effect model was performed in this case [32, 33], while the fixed-effect model was used when* P* > 0.05 and I^2^ < 50% [34]. Sensitivity analysis was conducted to explore, quantify, and control for sources of heterogeneity and stability of results across studies by excluding eligible studies by sequence. Begg's test [35], Egger's test [36] and the trim-and-fill method [37] were employed to identify the statistical significance of publication bias. All above statistical analyses were conducted by Stata (version 12.0, Stata Corp, College Station, TX, USA).

## Results

### Literature selection

At the beginning, a total of 914 records were identified through database searching, and eight additional records identified through other sources. After removal of the duplicates, 798 publications were identified for independent screening, of which 769 were deemed irrelevant on the basis of their title and abstract and 29 publications were eligible for full-text evaluation (inter-reviewer agreement, κ = 0.81). Of these articles, 22 were further excluded: 15 studies lack of control group, four studies without full text, two letter/review/meta-analysis and 1 study lack of primary clinical parameters (inter-reviewer agreement, κ = 0.92). Finally, seven RCTs met the eligibility criteria in this meta-analysis (Additional file 1: Figure S1). The excluded studies and reasons for exclusion were listed in Additional file 2: Table 1.

### Characteristics of the included studies

As shown in Table 1, main characteristics of the included trials are presented. They were published between 2009 and 2019. There was little variation in the number of participants enrolled in the 7 RCTs (24–36), reaching a total of 212 with mean age ranging between 35 and 60 years old. The studies were carried out in the following countries: Brazil (n = 2), Turkey (n = 3), China (n = 1) and Germany (n = 1). The percentages of female participants in the studies were summarized, which ranged between 40.0 and 100%. All studies reported the percentage of female participants. Five of these studies reported a duration of RA from 6 weeks to 14.9 years and two did not provide the duration information. The timing and frequency of follow-up varied amongst the 7 included studies, ranging from 1 to 6 months duration. Seven of the studies included outcomes of PD and BOP, six of PI, and three of GI and AL.

**Table 1:** Characteristics of eligible studies included in this meta-analysis

Author name, year	Location	Subjects	Gender (%Female)	Age	RA duration (years)	Follow-up time	Outcomes
Pinho et al. (2009) [42]	Brazil	Test: 15 Control: 15	60.0%	35–60	0.5–10	3, 6 months	PI, PD, BOP
Bıyıkoğlu et al. (2013) [43]	Turkey	Test: 15 Control: 15	Test: 60.0% Control: 40.0%	Test: 46.6 Control: 46.7	6.4	1,3,6 months	PI, PD, AL, BOP
Roman-Torres et al. (2015) [44]	Brazil	Test: 12 Control: 12	100%	Test: 45.4 Control: 46.8	10.0	3 months	PI, PD, BOP
Kurgan et al. (2016) [45]	Turkey	Test: 13 Control: 13	Test: 69.2% Control: 46.2%	Test: 48.5 Control: 41.4	NA	3 months	PI, GI, PD, BOP
Kurgan et al., (2017) [46]	Turkey	Test: 15 Control: 15	Test: 60.0% Control: 53.3%	Test: 49.3 Control: 42.1	NA	3 months	PI, GI, PD, AL, BOP
Zhao et al. (2018) [47]	China	Test: 18 Control: 18	Test: 77.8% Control: 77.8%	Test: 42.8 Control: 44.8	> 6 weeks	1 month	PI, GI, PD, BOP
Cosgarea et al. (2019) [40]	Germany	Test: 18 Control: 18	Test: 77.8% Control: 55.6%	Test: 51.6 Control: 43.6	14.9	3,6 months	PD, AL, BOP

AL, attachment loss; BOP, bleeding on probing; GI, gingival index; PD, probing depth; PI, plaque index; RA, rheumatoid arthritis

### Risk of bias within studies

**Fig. 1:**
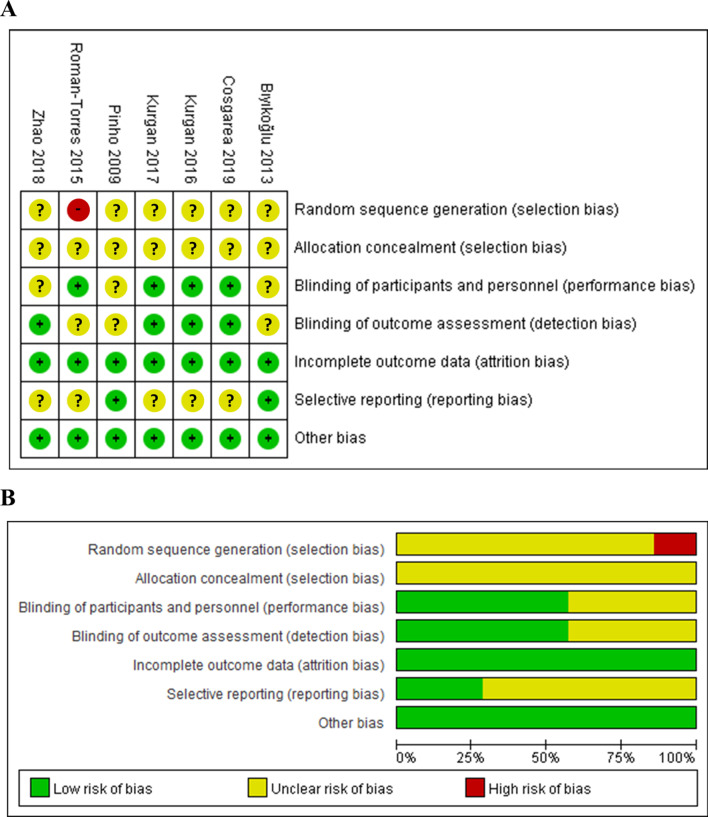
Risk of bias assessment for the studies included in the meta-analysis. **A** risk of bias summary; **B** risk of bias graph. (+): low risk of bias; (?): unclear risk of bias; (−): high risk of bias

The quality of evidence for each outcome was based on six domains: Selection bias, performance bias, detection bias, attrition bias, reporting bias and other bias. Results were presented graphically by study (Fig. 1A) and proportion chart of bias was set across all studies (Fig. 1B). It is noteworthy that only one of the studies was judged to be at high risk of selection bias, which was observed in Roman-torres 2015. Three studies had an unclear risk of bias in blinding of participants and personnel, 3 studies had an unclear risk of bias in blinding of outcome assessment, and 5 studies had an unclear risk of bias in selective reporting. In addition, with regard to allocation concealment, all studies showed unclear risk. Overall, only one trial showed a high risk of bias and the risk was unclear in the other studies.

### Meta-analysis of primary outcome

**Fig. 2:**
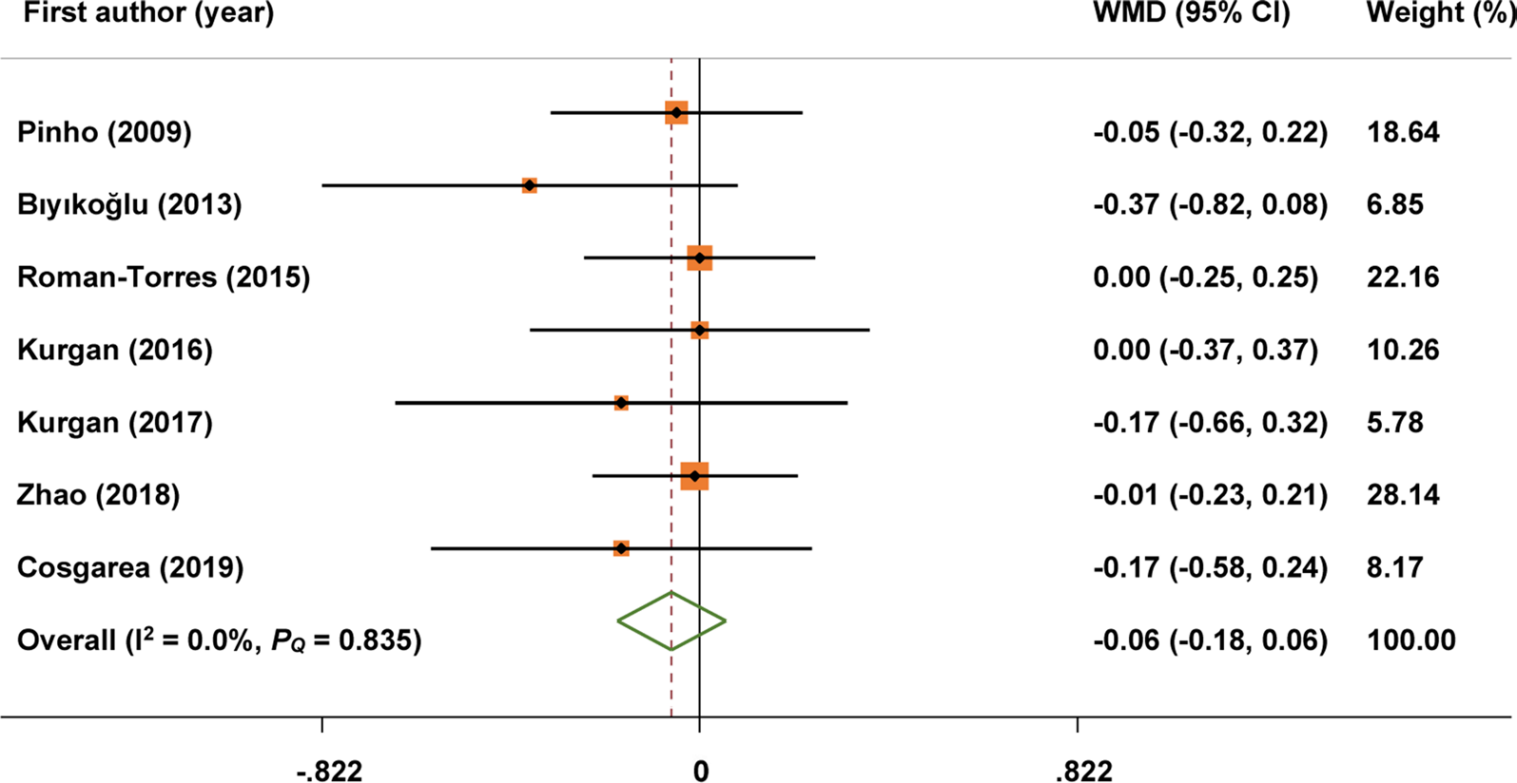
Forest plot of changes in probing depth

The primary outcome was reported in 7 studies. The change of the PD (MD: − 0.06; 95% CI: − 0.18, 0.06) was not statistically significant in periodontitis patients with RA compared with periodontitis control patients. No heterogeneity was observed for PD (I^2^ = 0.0%, *P* = 0.835), so a fixed-effect model was used (Fig. 2).

### Meta-analysis of secondary outcomes

**Fig. 3:**
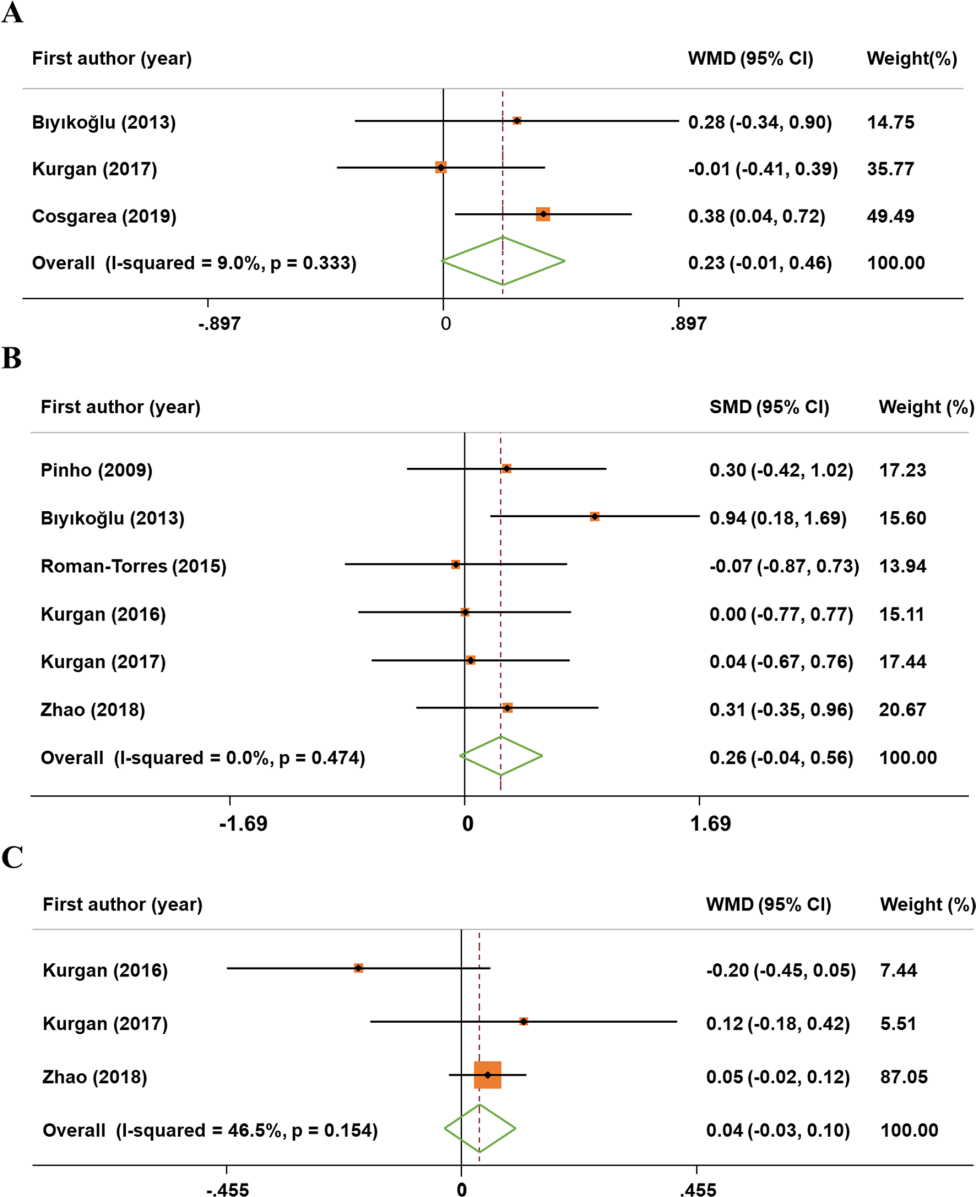
Forest plot of changes in attachment loss, plaque index and gingival index reduction. **A** attachment loss; **B** plaque index; **C** gingival index

The outcomes for AL, PI and GI were included in 3, 6 and 3 trails, respectively. Compared with periodontitis control patients, the changes of the AL (MD: 0.23; 95% CI: − 0.01, 0.46), PI (MD: 0.26; 95% CI: − 0.04, 0.56) and GI (MD: 0.04; 95% CI: − 0.03, 0.10) were not statistically significant in periodontitis patients with RA. No evidence of heterogeneity in AL (I^2^ = 9.0%, *P* = 0.333), PI (I^2^ = 0.0%, *P* = 0.474) and GI (I^2^ = 46.5%, *P* = 0.154) changes was found, so fixed-effect models were used (Fig. 3A–C).

**Fig. 4:**
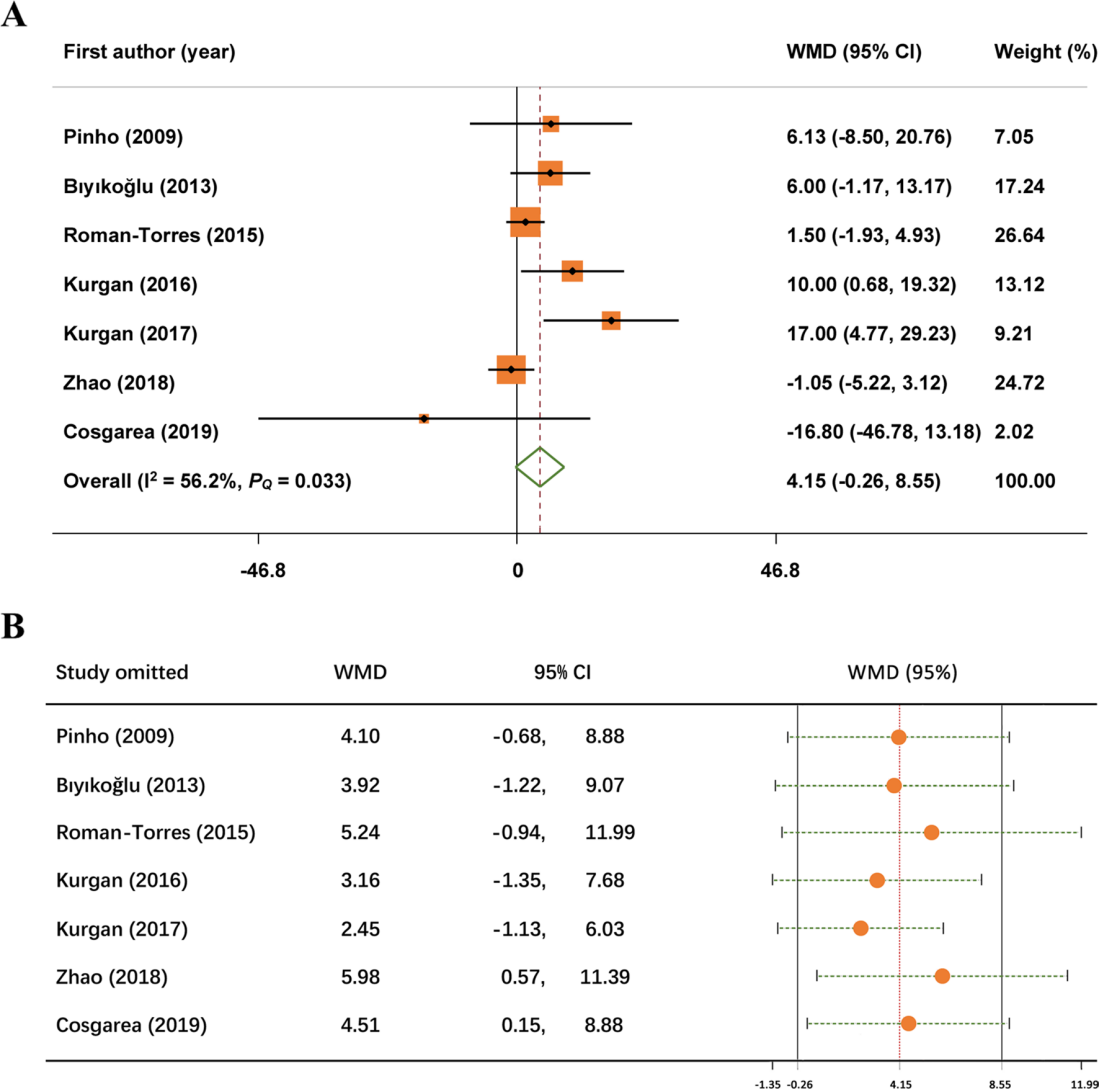
Forest plot and sensitivity analysis of changes in bleeding on probing reduction. **A** forest plot; **B** sensitivity analysis

As for the BOP changes, 7 studies have described this result. No statistically significant difference was showed between the two groups (MD: 4.15; 95% CI: − 0.26, 8.55). Notably, it was concluded that there was significant heterogeneity for BOP (I^2^ = 56.2%, *P* = 0.033) so a random-effect model was used (Fig. 4A).

### Sensitivity analysis

Sensitivity analyses were performed in order to assess the potential source of heterogeneity of BOP outcomes. We evaluated the influence of individual dataset on the pooled effect by omitting one study at a time and calculating the pooled outcomes for the remaining studies. However, the result of the sensitivity analysis concerning BOP indicated that no significant effect was observed after excluding any single study, suggesting that the result was relatively robust (Fig. 4B).

### Publication bias

Publication bias was evaluated through Begg's test, Egger's test and the trim-and-fill method. Begg's and Egger's test revealed that there was no publication bias for the changes of PI, GI, AL and BOP (*P* > 0.05), but Egger's test manifested that there was publication bias for PD (Egger’s test *P* = 0.04). The trim-and-fill analysis suggested no evidence of significant difference between the adjusted value and the original value of PD changes, but revealed a missing study for BOP changes. However, the adjusted value for BOP changes was also not significantly different from the original value (Table 2).

**Table 2:** Quantitative analysis of publication bias

Outcome	Studies trimmed/total studies	Trim-and-fill analysis		Begg’s test (*P*-value)	Egger’s test (*P*-value)
		MD	95%CI		
PD	0/7	− 0.06	− 0.18, 0.06	0.07	0.04
AL	0/3	0.23	− 0.01, 0.46	1.00	0.90
PI	0/6	0.26	− 0.04, 0.56	0.26	0.79
GI	0/3	0.04	− 0.03, 0.10	1.00	0.69
BOP	1/7	2.74	− 2.10, 7.58	0.76	0.34

AL, attachment loss; BOP, bleeding on probing; CI, confidence interval, GI, gingival index; MD, mean difference; PD, probing depth; PI, plaque index

### Subgroup analysis

To determine the potential influence of follow-up time on the clinical efficacy of SRP, we performed analyses separately for different follow-up time points. Compared with periodontitis control patients, the reduction of BOP (MD: 5.93; 95%CI: 0.28, 11.58) was significantly larger in periodontitis patients with RA at the 3rd month after SRP. Similarly, the changes of AL (MD: 0.36; 95% CI: 0.06, 065) and PI (MD: 0.60; 95% CI: 0.08, 1.13) of periodontitis patients with RA were slightly larger at the 6th month than periodontitis control patients (Table 3).

**Table 3:** Subgroup analysis according to different follow up time points

Follow-up time	No. of studies	Meta-analysis			Heterogeneity		Publicaton bias(*P*-value)	
		MD	95%CI	*P*-value	I^2^(%)	*P*-value	Begg’s test	Egger’s test
*PD*								
1mo	2	− 0.14	− 0.47, 0.20	0.43	50.5	0.16	1.00	NA
3mo	6	− 0.05	− 0.20, 0.09	0.43	0.0	0.59	0.13	0.05
6mo	3	− 0.14	− 0.35, 0.06	0.17	0.0	0.49	0.30	0.26
*AL*								
1mo	1	− 0.07	− 0.83, 0.69	0.86	NA	NA	NA	NA
3mo	3	− 0.02	− 0.24, 0.20	0.85	0.0	0.77	0.30	0.20
6mo	2	0.36	0.06, 0.65	0.02	0.0	0.78	1.00	NA
*PI*								
1mo	2	− 0.07	− 0.56, 0.42	0.78	64.1	0.10	1.00	NA
3mo	5	0.31	− 0.03, 0.64	0.08	17.0	0.31	0.46	0.59
6mo	2	0.60	0.08, 1.13	0.02	29.7	0.23	1.00	NA
*GI*								
1mo	1	0.05	− 0.02, 0.12	0.19	NA	NA	NA	NA
3mo	2	− 0.05	− 0.36, 0.26	0.76	61.2	0.11	1.00	NA
*BOP*								
1mo	2	− 0.77	− 4.35, 2.81	0.67	0.0	0.80	1.00	NA
3mo	6	5.93	0.28, 11.58	0.04	54.2	0.05	0.71	0.32
6mo	3	5.02	− 1.28, 11.32	0.12	6.0	0.35	0.30	0.28

AL, attachment loss; BOP, bleeding on probing; CI, confidence interval, GI, gingival index; MD, mean difference; PD, probing depth; PI, plaque index

## Discussion

To the best of our knowledge, available evidence was summarized in an effort to specifically estimate the clinical efficacy of SRP in periodontitis patients with RA for the first time of meta-analysis. Seven RCTs were included, and all the studies evaluated the changes associated to treatment of the periodontal inflammation, based on the measurement of different clinical indices and parameters (PI, GI, PD, AL and BOP). The findings from the present meta-analysis failed to find significant difference in the clinical efficacy of SRP between RA patients with periodontitis and patients with periodontitis alone. This provides evidence that RA does not affect the clinical efficacy of SRP in periodontitis.

All the indices/parameters failed to show significant difference between groups in this meta-analysis. As the primary result, the outcome of PD showed that the effects of SRP in patients with RA and periodontitis were almost consistent with those with periodontitis alone. The outcome of PI, GI, AL and BOP also demonstrated this conclusion. It is pertinent to mention that previous research demonstrated the application of the mechanical periodontal treatment as SRP could effectively improve periodontal parameters [38]. But whether this effect will change in the presence of RA is unclear. Thus, these findings confirmed that SRP is an effective treatment for periodontitis and the clinical benefits of SRP could not be affected by RA in periodontitis patients in this regard.

Recent report showed that the duration of RA is likely to have a significant impact on the association between RA and periodontitis [39]. What’s more, Qiao et al. declared that periodontitis might be more closely related to disease duration > 5 years of RA patients [27]. Also, a significantly higher clinical AL was found in moderately-to-highly active RA patients, compared to those in remission [39]. Unfortunately, information about the RA duration was not available in the studies enrolled in our meta-analysis. In addition, we did not confirm a significant difference of AL reduction in the two groups. We speculate that an imprecise duration of RA is likely to contribute to no difference in the SRP- related outcomes between groups. Clearly, this needs further investigation in well-designed studies taking this variable into account.

Cosgarea et al. observed a significant reduction in some clinical periodontal parameters within 3 and 6 months after treatment in patients with periodontitis and RA [40]. However comparisons of efficacy differences were not achieved. In our study, when stratified by the points of follow-up, the periodontitis patients with RA showed a higher BOP reduction at 3 months and an overall improvement for PI and AL at 6 months in comparison to periodontitis patients. However, it did not show significantly statistical differences in other parameters and follow-up time points. Besides, research reported no difference in clinical parameter outcomes when studying periodontal treatment effects of patients with low and high disease activity of RA [41]. Therefore, the difference in the efficacy of SRP between groups has not been well demonstrated by the outcomes for follow-up within 6 months after treatment.

There has long been an argue whether RA affect the outcome of SRP. However, the lack of comparative efficacy evidence may create uncertainty for physicians when encounter periodontitis with RA. This analysis can help provide clinicians with a framework when assessing periodontitis patients with RA in their clinical practice. Since evidence suggests that efficacy of SRP is not affected by RA, we recommend that at least routine periodontal treatment for periodontitis patients with RA is required. If a patient has periodontitis, then SRP can reasonably be offered on the grounds it will improve the clinical outcome of that patient. Similarly, if a patient has RA, that periodontitis patient is also likely to have their periodontal prognosis improved by SRP.

Although the study was designed seriously and data was processed carefully, we identified several limitations in this meta-analysis. First, the study is likely to lack the statistical power to detect differences between groups due to the limited number of studies and subjects. Second, 2 studies did not publish RA duration. As a result, potential confounding factors could lead to some bias in the outcomes. Third, there may also be some heterogeneity between the two groups in terms of gender and demographic data, which were not analyzed in the subgroup. Finally, some clinical criteria for disease assessing are not entirely consistent and tend to introduce non-differential misclassification of the two diseases, with a potential effect of driving the results towards no difference.

## Conclusions

Taken together, in spite of these limitations, we conclude that SRP is equally effective in RA as compared to non-RA periodontitis patients. It suggests RA does not affect the clinical efficacy of non-surgical periodontal therapy. These results could serve evidence-based practice. We are looking forward to additional scientific researches to elucidate the clinical efficacy of SRP in RA patients with periodontitis of various severities further.

## Supplementary Information

**Additional file 1: Figure S1**. Flow chart from identification of eligible studies to final inclusion.

**Additional file 2: Table S1**. Excluded studies and reasons for exclusion.

## Data Availability

The detailed data supporting the study are available upon reasonable request.

## Abbreviations

RA: Rheumatoid arthritis
SRP: Scaling and root planning
RCTs: Randomized controlled trials
SP: Severe periodontitis
PI: Plaque index
GI: Gingival index
AL: Attachment loss
PD: Probing depth
BOP: Bleeding on probing
PRISMA: Preferred Reporting Items for Systematic Reviews and Meta-Analyses
MeSH: Medical Subject Headings
MD: Mean difference
CI: Confidence intervals
