# Supplementary figures and images for: Effects of non-surgical periodontal therapy on periodontal clinical data in periodontitis patients with rheumatoid arthritis: a meta-analysis
Source: BMC Oral Health. 2021 Jul 10;21:340. doi: 10.1186/s12903-021-01695-w (PMC8272313; doi:10.1186/s12903-021-01695-w)

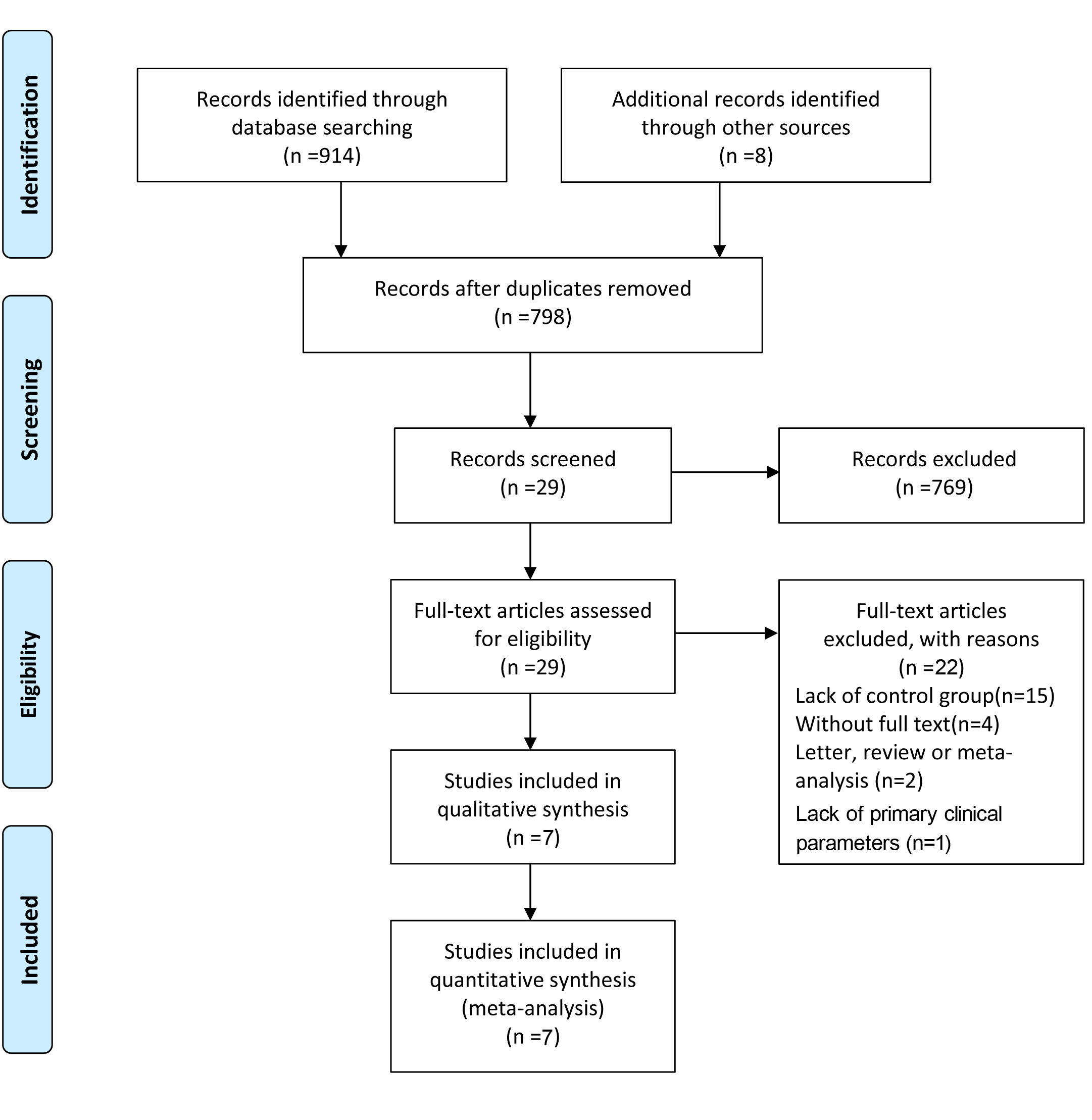

Supplement: Supplementary file 1 — Additional file 1: Figure S1. Flow chart from identification of eligible studies to final inclusion. [file 12903_2021_1695_MOESM1_ESM.png]
