# Supplementary material for: Effects of non-surgical periodontal therapy on periodontal clinical data in periodontitis patients with rheumatoid arthritis: a meta-analysis
Source: BMC Oral Health. 2021 Jul 10;21:340. doi: 10.1186/s12903-021-01695-w (PMC8272313; doi:10.1186/s12903-021-01695-w)
Supplement: Supplementary file 2 — Additional file 2: Table S1. Excluded studies and reasons for exclusion. [file 12903_2021_1695_MOESM2_ESM.docx]

**Supplementary Table 1. Excluded studies and reasons for exclusion.**

| **First author, year of publication** | **Reasons for exclusion** |
| --- | --- |
| Al-Katma et al., 2007 | Lack of control group  (patients with periodontitis alone) |
| Ortiz et al., 2009 |  |
| Sarika et al., 2012 |  |
| Erciyas et al., 2013 |  |
| Okada et al., 2013 |  |
| Monsarrat et al., 2013 |  |
| Khare et al., 2016 |  |
| Fazele et al., 2018 |  |
| Moeller et al., 2018 |  |
| Kaushal et al., 2019 |  |
| Anusha et al., 2019 |  |
| Monsarrat et al., 2019 |  |
| Białowąs et al., 2020 |  |
| Mariette et al., 2020 |  |
| Pandya et al., 2020 |  |
| de Smit et al., 2012 | Without full text |
| England et al., 2015 |  |
| Xiao et al., 2017 |  |
| S. A. Kusumo et al., 2018 |  |
| Marotte et al., 2020 | Letter, review or meta-analysis |
| Möller et al., 2020 |  |
| Yuce et al., 2017 | Lack of primary clinical parameters |
